# 10-Year Long-Term Outcomes of Robotic-Assisted Segmentectomy for Early-Stage Non-Small-Cell Lung Cancer

**DOI:** 10.3390/jcm14165608

**Published:** 2025-08-08

**Authors:** Monica Casiraghi, Riccardo Orlandi, Antonio Mazzella, Lara Girelli, Giovanni Caffarena, Matteo Chiari, Luca Bertolaccini, Giorgio Lo Iacono, Cristina Diotti, Claudia Bardoni, Patrick Maisonneuve, Lorenzo Spaggiari

**Affiliations:** 1 Department of Thoracic Surgery, European Institute of Oncology (IEO), IRCCS, 20141 Milan, Italy; antonio.mazzella@ieo.it (A.M.); lara.girelli@ieo.it (L.G.); giovanni.caffarena@ieo.it (G.C.); matteo.chiari@ieo.it (M.C.); luca.bertolaccini@gmail.com (L.B.); giorgio.loiacono@ieo.it (G.L.I.); cristina.diotti@ieo.it (C.D.); claudia.bardoni@ieo.it (C.B.); lorenzo.spaggiari@ieo.it (L.S.); 2 Department of Oncology and Hemato-Oncology, University of Milan, 20122 Milan, Italy; 3 Department of Thoracic Surgery, University of Milan, 20122 Milan, Italy; riccardo.orlandi@unimi.it; 4 Division of Epidemiology and Biostatistics, European Institute of Oncology (IEO), IRCCS, 20141 Milan, Italy; patrick.maisonneuve@ieo.it

**Keywords:** lung segmentectomy, robotic-assisted thoracic surgery, sublobar resection, long-term outcomes, lung cancer

## Abstract

**Objectives:** Robotic-assisted segmentectomy (RAS) has proven to be safe and feasible for early-stage lung cancer; nonetheless, its oncologic efficacy and long-term outcomes are still debated. We aimed to explore whether RAS could be an alternative to robotic-assisted lobectomy (RAL) in early-stage NSCLC, focusing on long-term outcomes such as 10-year cancer-specific survival (CSS), cumulative rate of relapse (RR), and local recurrence (LR). **Methods:** Patients undergoing RAS for early-stage NSCLC (clinical stage I) were analyzed from August 2007 to August 2023. A 1:3 propensity score-matched analysis was performed among patients undergoing RAL, based on demographic characteristics and pathological stage. Primary endpoints were CSS, RR, and LR. **Results:** A total of 40 patients undergoing RAS were retrospectively enrolled. After matching 120 patients undergoing RAL, no significant differences were found in postoperative complications, median operative time, or length of hospital stay. Patients undergoing RAS had comparable 10-year CSS (*p* = 0.90) and RR (*p* = 0.99) to those undergoing RAL, whereas 10-year of cumulative incidence of local recurrence (LR) was 11.0% (95% CI: 3.4–23.7%) for RAS patients and 2.8% (95% CI: 0.5–8.9%) for RAL patients (*p* = 0.08). Additionally, RAL provided a significantly higher number of N1 and N2 lymph node retrievals (*p* < 0.0001 and 0.06, respectively), as well as a higher number of N2 stations (*p* = 0.0001). **Conclusions:** Based on our experiences, even though RAS can ensure excellent long-term outcomes in selected cases of early-stage NSCLC, comparable to RAL, the local recurrence rate was higher in the RAS group.

## 1. Introduction

Lung cancer remains one of the leading causes of cancer-related mortality worldwide, with non-small-cell lung cancer (NSCLC) accounting for approximately 85% of all lung cancer cases [1]. For patients with early-stage NSCLC, surgical resection has always been considered the treatment of choice, offering the best chance for long-term survival [2]. Traditionally, lobectomy has been the gold standard for the surgical management of this tumor [3], but thanks to an attentive screening program, and a more frequent identification of small lung tumors, the thoracic surgery community has been pushed into developing less invasive resections and surgical approaches.

Recent evidence from the JCOG0802/WJOG4607L and CALGB 140503 trials has established lung segmentectomy as a valid alternative to lobectomy in selected patients with NSCLC smaller than 2 cm, demonstrating that segmentectomy can provide comparable long-term oncologic outcomes to lobectomy, without significant compromise in pulmonary function [4,5]. These findings have established sublobar resection, particularly segmentectomy, as a safe and oncologically sound option for small, peripheral NSCLC. However, technical complexity—especially in identifying and dividing segmental structures while ensuring adequate oncologic margins—poses challenges in both open and minimally invasive surgery.

In recent years, the robotic-assisted approach to segmentectomy (RAS) has emerged as a promising evolution in thoracic surgery. Robotic systems offer several advantages over conventional video-assisted thoracoscopic surgery (VATS), including enhanced three-dimensional visualization, increased instrument articulation, tremor filtration, and improved ergonomics, which are particularly beneficial when performing precise anatomical dissections in segmentectomy [6,7].

The use of robotics is especially advantageous in complex segmentectomies—such as apical or basilar segments—where clear identification of intersegmental planes and lymphadenectomy could be more challenging. A systematic review and meta-analysis by Zhang et al. compared robotic and VATS approaches for both lobectomy and segmentectomy, reporting reduced blood loss, lower conversion rates to thoracotomy, and shorter hospital stays in the robotic group, with similar oncologic outcomes [8].

Despite these promising short-term outcomes, long-term data specifically evaluating oncologic efficacy and recurrence rates of RAS remain limited. Nevertheless, retrospective studies have shown comparable oncologic outcomes between robotic and VATS segmentectomy, particularly in early-stage disease [9]. Ongoing prospective registries and future randomized studies will be essential to further validate RAS as a standard approach for early-stage NSCLC.

This study aims to explore whether RAS can be an alternative to robotic-assisted lobectomy (RAL) in early-stage NSCLC, with a specific focus on long-term outcomes, including 10-year cancer-specific survival (CSS), cumulative rate of relapse (RR), and local recurrence (LR).

## 2. Materials and Methods

A retrospective analysis of 1040 patients who underwent robotic anatomical lung resection for early-stage NSCLC between August 2007 and August 2023 was performed at the European Institute of Oncology in Milan. Forty patients underwent RAS, and each patient was retrospectively matched with three patients undergoing RAL over the same period, by propensity score, optimal fixed ratio matching (1:3) based on demographic characteristics (age, sex), pathological features (tumor size and grade, histology, tumor site), and pathological stage, following 8th TNM staging system.

Inclusion criteria were clinical stage IA and IB NSCLC (less than 4 cm in size, N0 and no evident pleural involvement) according to the 8th edition of the TNM staging system in lung cancer; intent-to-treat anatomical segmentectomy or lobectomy with radical lymphadenectomy, performed by robotic approach; no preoperative induction therapy; no previous history of concurrent malignant disease or other previous primary lung cancer. We excluded from the study all patients with one or more of the following conditions: histology other than NSCLC; incomplete preoperative staging; incomplete lymphadenectomy; or anatomical resection other than anatomical segmentectomy or lobectomy.

The decision to perform anatomical segmentectomy or lobectomy was based on the patient’s fitness for surgery (respiratory function, previous lung surgery) and the surgeon’s choice.

All segmentectomies were “simple” segmentectomies following the definition proposed by Handa et al. [9].

Data were collected retrospectively from patients’ medical records, including demographic information, operative details, postoperative outcomes, and long-term follow-up data.

The study was approved by the Institutional Review Board (UID 4367) of our Institution, and all patients provided informed consent to undergo surgery and for their data to be used in research.

### 2.1. Preoperative Assessment

Preoperative staging was based on a total-body computed tomography scan (CT scan) and positron emission tomography with fluorodeoxyglucose (PET) scan. Whenever possible, suspected lymph node involvement was verified with endobronchial ultrasound-guided transbronchial needle aspiration (EBUS-TBNA) or mediastinoscopy. Staging and functional exams were always performed within 5 weeks before surgery.

### 2.2. Surgical Procedures

All patients underwent segmentectomy or lobectomy via robotic approach along with systematic lymph node dissection, under general anesthesia and single-lung ventilation. The four-arm robotic approach, as described elsewhere [10], was applied. Briefly, a 3 cm utility incision was made in the fourth or fifth intercostal space, anteriorly, along with three additional 8 mm ports: one for the camera in the seventh or eighth intercostal space along the mid-axillary line, and two others at the seventh or eighth intercostal space in the posterior axillary line and in the auscultatory triangle at the sixth intercostal space, respectively. Until 2015, da Vinci S and SI systems (Intuitive Surgical Inc., Sunnyvale, CA, USA) were applied, whereas the da Vinci Xi system was introduced thereafter. Pulmonary arteries, veins, and bronchi were resected either with a mechanical stapler (EndoGIA 30 vascular and 30 or 45 parenchyma) or with a robotized stapler (EndoWrist 30 or 45 vascular and parenchyma). The segmental plane was completed with inflation of the lobe. Patients with N1 involvement or with positive margins on frozen section underwent lobectomy and were excluded from the analysis. We do not perform frozen sections on N2 unless they are suspected during surgery.

Operating time was measured from the first incision to the final closure of the wound.

### 2.3. Postoperative Follow-Up

Patients in both groups were followed with a physical examination, chest X-ray, and blood tests 1 month after surgery. Subsequent follow-up consisted of a physical exam plus chest and upper abdomen CT scans every 4 months for 3 years, then every 6 months until the fifth year, and annually thereafter. After 10 years, follow-up was updated by telephone, through requests sent to local registries, and information contained in the institutional personal dossier.

Recurrences in the hilar/mediastinal nodal area or lung tissue near the previous resection were recorded as local. New pulmonary nodules or pleural lesions on the same or opposite side were classified as regional, while any lesions found outside the thoracic cavity were considered distant metastases.

Postoperative complications were defined as any complication occurring within 30 days after surgery and were categorized using the Thoracic Morbidity and Mortality classification system [11]. Postoperative death was defined as mortality occurring within 30 days after surgery or during hospitalization.

### 2.4. Statistical Analysis

Absence of normality was confirmed using the Shapiro–Wilk test for all continuous variables considered in the analysis, which were described as median and range and compared using a non-parametric median test. Categorical data were expressed as numbers and frequencies and were compared using the Chi-squared test. CSS was defined as the time from the date of surgery to the date of death due to lung cancer; instead, relapse was defined as the occurrence of local, regional, or distant lesions related to the treated lung cancer. Deaths from unrelated causes were treated as competing events. Survival outcomes, including CSS and RR, were analyzed using the Kalbfleisch–Prentice method [12] and compared by Gray’s test [13]. Statistical significance was defined as a *p*-value of less than 0.05. Analysis was performed using SAS software v. 9.4 (SAS Institute, Cary, NC, USA).

## 3. Results

Forty patients who underwent RAS and fulfilled the inclusion criteria were propensity score-matched (1:3) with 120 patients undergoing RAL. The median age of patients in the RAS group was 64.5 years (range, 50–85 years), compared to 66 years (range, 43–83 years) in the RAL group (*p* = 0.69). In the RAS and RAL groups, 22 patients (55.0%) and 53 patients (44.2%), respectively, were male (*p* = 0.23).

In the segmentectomy group, 17 patients had S6 segmentectomy, 17 patients underwent apico-dorsal segmentectomy (left S1 + S2 + S3), three patients underwent lingulectomy (left S4 + S5), and three patients had a right apico-ventral segmentectomy (right S1 + S2).

Adenocarcinoma was the most common histology in both cohorts (87.5% and 87.5%, respectively; *p* = 1.00), and most tumors were graded as G2 (60.0% and 68.3%, respectively; *p* = 0.63). The median tumor size was 12.5 (6–34) mm in the RAS group, compared to 15 (6–32) mm in the RAL group (*p* = 0.64). The majority of tumors were at stage I of the disease in both groups (90.0% and 96.7%, respectively; *p* = 0.17).

The characteristics of patients, operative details, and pathological features for matched RAS and RAL cohorts are detailed in Table 1.

Only one patient (2.5%) in the RAS group was converted to thoracotomy for pleural adhesions. In contrast, three patients (2.5%) in the RAL group were converted to open surgery (one for bleeding and two for pleural adhesions).

### 3.1. Short-Term Outcomes

The short-term outcomes of the matched RAS and RAL cohorts are presented in Table 2.

The median operative time for RAS was 159 (95–224) min, which was slightly shorter but not statistically significant compared to the median operative time for RAL, 167 (70–348) min (*p* = 0.27). The median length of hospital stay was shorter in the RAS group, with a median of 4 days (range, 3–21 days), compared to 5 days (range, 3–35 days) in the RAL group (*p* = 0.10). Notably, none of the patients in the RAS cohort experienced a postoperative Intensive Care Unit (ICU) stay, whereas 15 (12.5%) patients in the RAL cohort did (*p* = 0.02). The 30-day mortality rate was 0% in both groups, with a 30-day morbidity rate of 25% in the RAS group and 23.3% in the RAL group (*p* = 0.83). In particular, into the RAS cohort, 2 (5.0%) patients experienced major complications such as severe pulmonary embolism in 1 case (grade IV) and sputum retention requiring bronchoscopy in another patient (grade IIIa); 8 (20.0%) patients experienced minor complications such as pulmonary atelectasis in 2 cases (grade I), wound hematoma in 1 case (grade II), pleural effusion in 1 patient (grade II), two atrial fibrillation (grade II), and 2 cases of prolonged air leak (grade II).

In terms of lymph node harvesting, the RAS group had significantly lower median numbers of N2 nodal stations sampled compared to the RAL group: 2 [0–5] and 3 [1–5], respectively, p = 0.0001, whereas the median number of N1 stations harvested was overlapping between the two groups (RAS 2 (0–4) vs. RAL 2 (0–5); *p* = 0.18). Additionally, the median number of lymph nodes retrieved was significantly lower in the RAS group compared to the RAL group, both in station N1 and N2 (*p* < 0.0001 and *p* = 0.06, respectively) (Table 2).

### 3.2. Long-Term Outcomes

The overall median follow-up was 60 months [range 1 to 190]. In particular, the median follow-up for the RAS group was 118 months [range 6 to 190].

**Figure 1:**
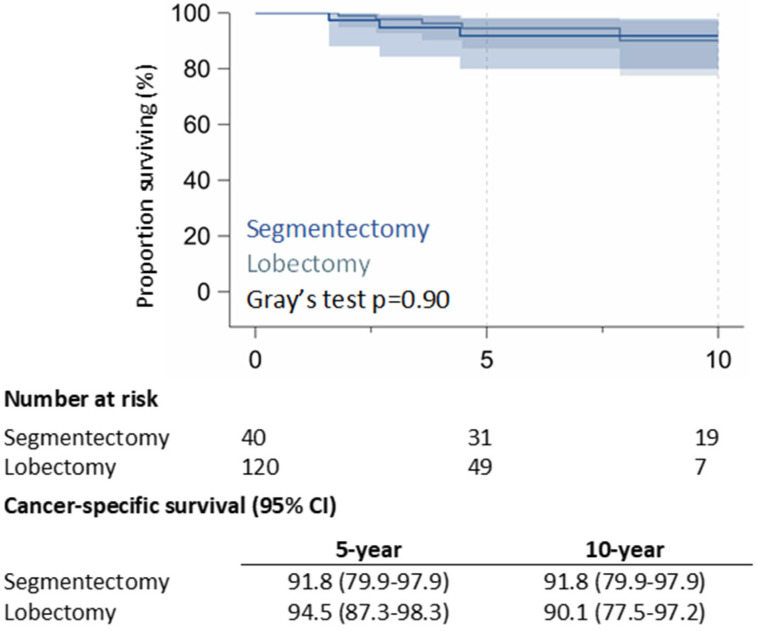
Cancer-specific survival.

**Figure 2:**
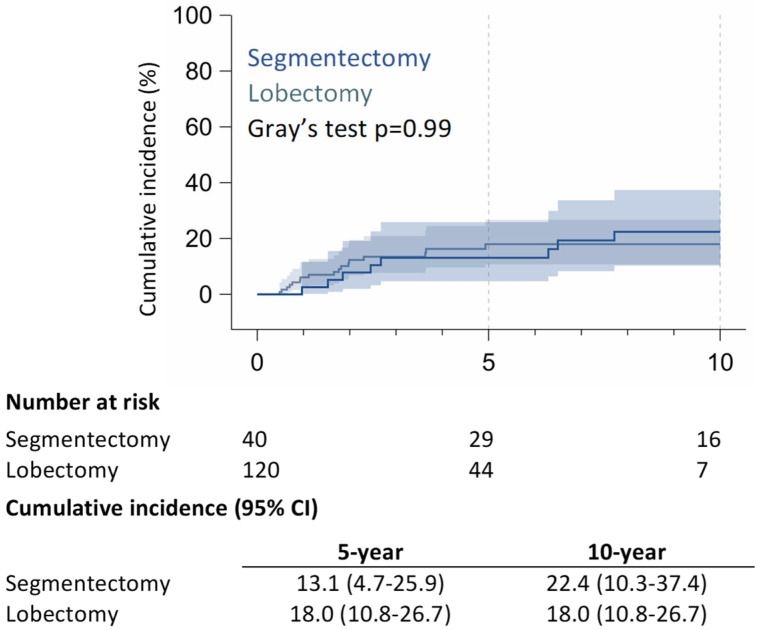
Cumulative incidence of relapse and cancer-specific survival.

The 10-year CSS rate was 91.8% (95% CI: 79.9–97.9%) in the RAS group and 90.1% (95% CI: 77.5–97.2%) in the RAL group (*p* = 0.90) (Figure 1); 5-year of RR was 13.1% (95% CI: 4.7–25.9%) in the RAS group and 18.0% (95% CI: 10.8–26.7%) in the RAL group; 10-year of RR was 22.4% (95% CI: 10.3–37.4%) for RAS patients and 18.0% (95% CI: 10.8–26.7%) for RAL patients (*p* = 0.99) (Figure 2).

**Figure 3:**
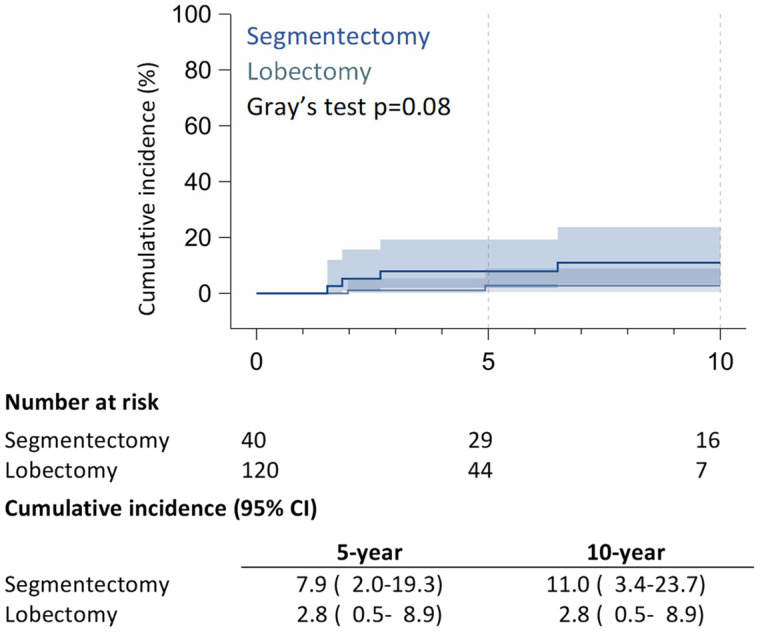
Cumulative incidence of local recurrence (LR).

Within the RAS cohort, 8 (20%) patients had recurrences: 5 (12.5%) patients experienced local recurrences (LRs) (4 on lung parenchyma and one on ipsilateral mediastinal nodes), 2 (5%) patients developed regional recurrence on contralateral lung parenchyma, and 1 (2.5%) patient developed distant recurrence on the bone. Within the RAL matched group, 17 (14.2%) patients experienced recurrences: 2 (1.7%) patients had local recurrence with hilar lymph node relapses, 8 (6.7%) patients presented regional recurrences (two ipsilateral pleural lesions and six contralateral pulmonary nodules), and 7 (5.8%) patients had distant metastasis, mainly brain recurrences; 5-year of LR was 7.9% (95% CI: 2.0–19.3%) in the RAS group and 2.8% (95% CI: 0.5–8.9%) in the RAL group; 10-year of LR was 11.0% (95% CI: 3.4–23.7%) for RAS patients and 2.8% (95% CI: 0.5–8.9%) for RAL patients (*p* = 0.08) (Figure 3).

## 4. Discussion

Lung segmentectomy has recently been proven to be a valid alternative to lobectomy in selected patients with NSCLC < 2 cm [4,5]. In the early 2010s, the first reports of experiences in RAS appeared in the literature, yielding encouraging results [14,15]. Since then, several studies have consecutively demonstrated its safety and feasibility [16,17,18,19], which explains the increasing use of the robotic approach for segmentectomies [20,21,22]. The short-term outcomes of RAS are encouraging and not only comparable to those provided by VATS or open surgery but also superior [16,19,23,24]. Recently, RAS has been demonstrated to be a feasible alternative to RAL for conserving lung function in patients with respiratory-compromised lung cancer [25], but achieving favorable oncological outcomes remains the top priority for thoracic surgeons. The NCCN guidelines [2] emphasized that both VATS and RATS were viable options for patients without anatomical or surgical contraindications, provided that the fundamental oncologic principles and dissection techniques of thoracic surgery are not compromised. Nonetheless, the oncologic efficacy and long-term outcomes of this procedure remain under investigation, primarily due to the limited median follow-up, which, in the literature, currently does not exceed 5 years [16,26]. Recent high-quality randomized controlled trials have significantly influenced the surgical management of early-stage non-small-cell lung cancer (NSCLC). The JCOG0802/WJOG4607L trial, a multicenter, open-label, phase III non-inferiority study conducted in Japan, compared segmentectomy to lobectomy in patients with small (≤2 cm), peripheral clinical stage IA NSCLC. The trial enrolled 1106 patients and demonstrated that segmentectomy was non-inferior to lobectomy in terms of overall survival, with a 5-year overall survival rate of 94.3% in the segmentectomy group versus 91.1% in the lobectomy group. Importantly, the proportion of patients with local relapse was almost double for segmentectomy compared to lobectomy (10.5% vs. 5.4%, *p* = 0.0018) [4].

Similarly, the CALGB 140503 trial, a phase III randomized controlled study conducted in North America, evaluated sublobar resection (either wedge resection or segmentectomy) versus lobectomy in patients with peripheral NSCLC ≤ 2 cm in diameter. Among the 697 patients analyzed, sublobar resection was found to be non-inferior to lobectomy for disease-free survival, with 3-year DFS rates of 63.6% for sublobar resection and 64.1% for lobectomy. Overall survival and recurrence rates were also comparable, further supporting the use of limited resection in properly selected patients [5]. Thus, both trials confirmed lung segmentectomy as a valid alternative to lobectomy in selected patients with peripheral NSCLC smaller than 2 cm, in terms of oncological outcome, but without any consistent data on the surgical approach (VATS vs. RATS vs. open approach). Even in our study, RAS offers excellent long-term outcomes for selected patients with early-stage NSCLC, comparable to RAL; the 10-year CSS rate was as high as 92% in the RAS group, underscoring the oncologic efficacy of RAS in achieving durable disease control in selected cases. In particular, in the 5 years of follow-up, our 5-year survival rate seems to be in line with those reported in the literature, even if slightly worse than the 100% 5-year OS found by Zhou and colleagues [16], who included only stage IA, but better than the 55% 5-year CSS reported by Nguyen and colleagues [26], who included also patients with pN+ or pT2, as in our study. However, it is essential to note that the rate of LR was significantly higher in the RAS group (12.5%) compared to the RAL group (1.7%) (*p* = 0.08).

The lower rate of ICU stays observed in the RAS group compared to the RAL group suggests a favorable safety profile, likely attributable to the less extensive nature of segmentectomy compared to lobectomy.

The morbidity rates were comparable between the RAS and RAL groups, indicating that RAS does not increase the risk of postoperative complications compared to RAL. These findings align with the results reported in the literature [24]. However, the 30-day morbidity rate observed in our cohort appears to be slightly higher than that reported in the literature, which ranges from 10% to 24% [17]. Nonetheless, the great majority of our postoperative complications were minor, not requiring specific treatment. These findings support the growing adoption of RAS as a viable surgical option for selected patients with early-stage NSCLC, particularly those for whom preservation of lung function is a priority.

One of the key differences observed between RAS and RAL was the extent of lymph node dissection. RAS resulted in significantly lower lymph node retrieval and station sampling, which is consistent with the less extensive nature of segmentectomy. However, this lower nodal retrieval did not result in worse long-term survival or higher relapse rates, suggesting that the oncologic control achieved with RAS is adequate despite the lower extent of lymph node dissection.

The ability of RAS to achieve comparable survival outcomes with less extensive dissection may offer significant benefits in terms of reduced surgical morbidity.

The findings of this study highlight the potential of RAS to combine the benefits of minimally invasive surgery with effective oncologic control, making it an attractive option for selected patients with early-stage disease. This could be particularly true in the case of complex segmentectomies, involving the resection of multiple or anatomically challenging pulmonary segments, which represent a technically demanding subset of sublobar lung resections. Unlike simple segmentectomies—which typically involve well-defined, easily accessible segments—complex procedures require meticulous dissection of intricate broncho-vascular structures and precise identification of intersegmental planes, and definitely robotic approach in synergy with new tools such as a three-dimensional (3D) modeling and virtual reality, could significantly enhance preoperative planning facilitating the accurate delineation of intersegmental planes and optimizing resection margins while preserving healthy lung parenchyma. The integration of these technologies in clinical practice will contribute to reduced operative time, decreased intraoperative complications, and improved surgeon preparedness. Consequently, such advanced planning tools could hold the potential to improve both oncologic and functional outcomes in segmentectomy procedures.

In the present study, complex segmentectomies were not included, as these procedures have not yet been performed at our institution. This decision is based on concerns regarding the oncological risk of inadequate resection margins, which may lead to increased rates of local recurrence if the surgery is not meticulously planned and executed. Furthermore, given the absence of clear clinical benefits to the patient at this time, we have prioritized simpler segmentectomies to ensure both oncologic safety and optimal functional outcomes. The retrospective nature of this study and its relatively small sample size are notable limitations that must be taken into account when interpreting these results. These include selection bias, as patients selected for robotic surgery may have more favorable tumor characteristics or better baseline functional status, and institutional bias, since we are a high-volume center with significant expertise in robotic surgery. Moreover, confounding variables such as surgeon experience, extent of lymphadenectomy, and postoperative care protocols are often uncontrolled, limiting the generalizability of these findings. Furthermore, although propensity score matching was employed to reduce selection bias, it was based on a limited set of available characteristics, and the possibility of residual confounding cannot be completely ruled out. Also, the lack of complex segmentectomies and the long period considered in the study, influenced by technical and technological developments without taking into account the learning curve, may have introduced an inherited selection bias.

Prospective randomized trials are needed to confirm the long-term outcomes of this study and to explore further the comparative efficacy of RAS and RAL in different patient populations. Additionally, while the study provides valuable insights into the 10-year outcomes of RAS, a longer follow-up is necessary to fully assess the durability of these outcomes.

## 5. Conclusions

RAS is an effective and safe surgical option for selected patients with ES-NSCLC, offering good long-term outcomes with 10-year CSS comparable to RAL. However, lymph node dissection was less extensive in the RAS group, and the incidence of LR was definitely higher compared to RAL. Future research should focus on larger, prospective studies to validate these findings and to explore the potential benefits of RAS in broader cohorts. As robotic surgical technology continues to advance, it is likely to play an increasingly important role in the management of early-stage NSCLC, offering patients less invasive yet effective treatment options.

## Figures and Tables

**Table 1:** Patients’ characteristics, surgical details, and pathological features among matched RAS and RAL cohorts.

	Segmentectomy *	Lobectomy	*p*-Value
	N = 40	N = 120	
Age			0.69
Median [range] IQR	64.5 [50–85] 62–72	66 [43–83] 62–70	
<60	6 (15.0)	14 (11.7)	
60–69	22 (55.0)	75 (62.5)	
70+	12 (30.0)	31 (25.8)	
Sex			0.23
Men [43–83]	22 (55.0)	53 (44.2)	
Women	18 (45.0)	67 (55.8)	
Conversion			1.00
No	39 (97.2)	117 (97.5)	
Yes	1 (2.5)	3 (2.5)	
Side			0.46
Right	14 (35.0)	50 (41.7)	
Left	26 (65.0)	70 (58.3)	
Site			0.31
Upper	24 (60.0)	61 (50.8)	
Lower	16 (40.0)	59 (49.2)	
Histology			1.00
Adenocarcinoma	35 (87.5)	105 (87.5)	
Squamous	2 (5.0)	6 (5.0)	
Other	3 (7.5)	9 (7.5)	
Tumor grade			0.63
G1	11 (27.5)	26 (21.7)	
G2	24 (60.0)	82 (68.3)	
G3	5 (12.5)	12 (10.0)	
Tumor size			0.64
Median [range] IQR	12.5 [6–34] 8–17	15 [6–32] 11–18	
≤15 mm	28 (70.0)	81 (67.5)	
>15–30 mm	10 (25.0)	36 (30.0)	
>30–40 mm	2 (5.0)	3 (2.5)	
Clinical stage			0.007
1a	26 (65.0)	44 (36.7)	
1b	10 (25.0)	50 (41.7)	
1c	1 (2.5)	19 (15.8)	
2a	3 (7.5)	7 (5.8)	
Pathological stage			0.17
Stage I	36 (90.0)	116 (96.7)	
Stage II	3 (7.5)	2 (1.7)	
Stage III	1 (2.5)	2 (1.7)	
Adjuvant therapy			1.00
No	39 (97.5)	115 (95.8)	
Yes	1 (2.5)	5 (4.2)	

Matched for age, sex, histology (exact), pT, pN, tumor size, tumor grade, tumor side, tumor site, using propensity score. * Segmentectomy included 17 S6 segmentectomies, 17 left apico-posterior segmentectomies, three lingulectomies, and three right apico-dorsal segmentectomies. IQR: Interquartile range.

**Table 2:** Short-term outcomes and lymph node retrieval of the matched RAS and RAL cohorts.

	Segmentectomy	Lobectomy	*p*-Value
	N = 40	N = 120	
Operative time			
Median [range] IQR	159 [95–224] 150–182	167 [70–348] 138–193	0.27
N1 resected			
Median [range] IQR	4 [0–17] 3–7	9 [0–27] 6–13	**<0.0001**
N2 resected			
Median [range] IQR	3 [0–15] 2–5	5 [1–32] 3–7	0.06
Total N1 + N2 resected			
Median [range] IQR	9 [1–20] 6–13	15 [4–44] 11–19	**0.0004**
N1 stations			
Median [range] IQR	2 [0–4] 2–3	2 [0–5] 2–3	0.18
N2 Stations			
Median [range] IQR	2 [0–5] 1–3	3 [1–5] 2–3	**0.0001**
Total N1 + N2 stations			
Median [range] IQR	4 [1–7] 3–5	5 [2–8] 4–6	**0.0004**
Upstaging			
pN0	39 (97.5)	138 (98.3)	
pN+	1 (2.5)	2 (1.6)	1.00
Length of stay			
Median [range] IQR	4 [3–21] 3–5.5	5 [3–35] 4–6	0.10
ICU			
No	40 (100.0)	105 (87.5)	
Yes	0 (0.0)	15 (12.5)	**0.02**
Postoperative complications			
No	30 (75.0)	92 (76.7)	
Yes	10 (25.0)	28 (23.3)	0.83
Minor	8 (20.0)	25 (20.8)	
Major	2 (5.0)	3 (2.5)	0.59

Bold *p*-values indicate a statistically significant difference with a *p*-value less than 0.05. IQR: interquartile range.

## Data Availability

The original contributions presented in this study are included in the article.

## Abbreviations

The following abbreviations are used in this manuscript:

CSS	Cancer-specific survival
RR	Rate of relapse
ICU	Intensive care unit
NSCLC	Non-small-cell lung cancer
RAL	Robotic-assisted lobectomy
RAS	Robotic-assisted segmentectomy
CI	Confidence interval
SD	Standard deviation
LR	Local recurrence

## References

[1] Bray F., Laversanne M., Sung H., Ferlay J., Siegel R.L., Soerjomataram I., Jemal A. (2024). Global cancer statistics 2022: GLOBOCAN estimates of incidence and mortality worldwide for 36 cancers in 185 countries. CA Cancer J. Clin..

[2] (2024). NCCN Clinical Practice Guidelines in Oncology (NCCN Guidelines^®^): Non-Small Cell Lung Cancer, Version 9.2024. https://www.nccn.org/professionals/physician_gls/pdf/nscl.pdf.

[3] Ginsberg R.J., Rubinstein L.V. (1995). Randomized trial of lobectomy versus limited resection for T1 N0 non-small cell lung cancer. Lung Cancer Study Group. Ann. Thorac. Surg..

[4] Saji H., Okada M., Tsuboi M., Nakajima R., Suzuki K., Aokage K., Aoki T., Okami J., Yoshino I., Ito H. (2022). Segmentectomy versus lobectomy in small-sized peripheral non-small-cell lung cancer (JCOG0802/WJOG4607L): A multicentre, open-label, phase 3, randomised, controlled, non-inferiority trial. Lancet.

[5] Altorki N., Wang X., Kozono D., Watt C., Landrenau R., Wigle D., Port J., Jones D.R., Conti M., Ashrafi A.S. (2023). Lobar or Sub-lobar Resection for Peripheral Stage IA Non-Small-Cell Lung Cancer. N. Engl. J. Med..

[6] Park B.J., Flores R.M., Rusch V.W. (2006). Robotic assistance for video-assisted thoracic surgical lobectomy: Technique and initial results. J. Thorac. Cardiovasc. Surg..

[7] Mattioni G., Palleschi A., Mendogni P., Tosi D. (2023). Approaches and outcomes of Robotic-Assisted Thoracic Surgery (RATS) for lung cancer: A narrative review. J. Robot. Surg..

[8] Zhang J., Feng Q., Huang Y., Ouyang L., Luo F. (2022). Updated Evaluation of Robotic- and Video-Assisted Thoracoscopic Lobectomy or Segmentectomy for Lung Cancer: A Systematic Review and Meta-Analysis. Front. Oncol..

[9] Handa Y., Tsutani Y., Mimae T., Tasaki T., Miyata Y., Okada M. (2019). Surgical Outcomes of Complex Versus Simple Segmentectomy for Stage I Non-Small Cell Lung Cancer. Ann. Thorac. Surg..

[10] Casiraghi M., Cara A., Mazzella A., Girelli L., Lo Iacono G., Uslenghi C., Caffarena G., Orlandi R., Bertolaccini L., Maisonneuve P. (2024). 1000 Robotic-assisted lobectomies for primary lung cancer: 16 years single center experience. Lung Cancer..

[11] Ivanovic J., Al-Hussaini A., Al-Shehab D., Threader J., Villeneuve P.J., Ramsay T., Maziak D.E., Gilbert S., Shamji F.M., Sundaresan R.S. (2011). Evaluating the reliability and reproducibility of the Ottawa Thoracic Morbidity and Mortality classification system. Ann. Thorac. Surg..

[12] Xia F., Ning J., Huang X. (2018). Empirical Comparison of the Breslow Estimator and the Kalbfleisch Prentice Estimator for Survival Functions. J. Biom. Biostat..

[13] Dignam J.J., Kocherginsky M.N. (2008). Choice and interpretation of statistical tests used when competing risks are present. J. Clin. Oncol..

[14] Dylewski M.R., Ohaeto A.C., Pereira J.F. (2011). Pulmonary resection using a total endoscopic robotic video-assisted approach. Semin. Thorac. Cardiovasc. Surg..

[15] Pardolesi A., Park B., Petrella F., Borri A., Gasparri R., Veronesi G. (2012). Robotic anatomic segmentectomy of the lung: Technical aspects and initial results. Ann. Thorac. Surg..

[16] Zhou Q., Huang J., Pan F., Li J., Liu Y., Hou Y., Song W., Luo Q. (2020). Operative outcomes and long-term survival of robotic-assisted segmentectomy for stage IA lung cancer compared with video-assisted thoracoscopic segmentectomy. Transl. Lung Cancer Res..

[17] Perroni G., Veronesi G. (2020). Robotic segmentectomy: Indication and technique. J. Thorac. Dis..

[18] Cerfolio R.J., Watson C., Minnich D.J., Calloway S., Wei B. (2016). One Hundred Planned Robotic Segmentectomies: Early Results, Technical Details, and Preferred Port Placement. Ann. Thorac. Surg..

[19] Zhang Y., Chen C., Hu J., Han Y., Huang M., Xiang J., Li H. (2020). Early outcomes of robotic versus thoracoscopic segmentectomy for early-stage lung cancer: A multi-institutional propensity score-matched analysis. J. Thorac. Cardiovasc. Surg..

[20] Haruki T., Kubouchi Y., Kidokoro Y., Matsui S., Ohno T., Kojima S., Nakamura H. (2024). A comparative study of robot-assisted thoracoscopic surgery and conventional approaches for short-term outcomes of anatomical segmentectomy. Gen. Thorac. Cardiovasc. Surg..

[21] Leung A., Akhmerov A., Justo M., Fong A., Mahfoozi A., Soukiasian H.J., Imai T.A. (2023). Trends in segmentectomy for the treatment of stage 1A non-small cell lung cancers: Does the robot have an impact?. Am. J. Surg..

[22] Kneuertz P.J., Zhao J., D’Souza D.M., Abdel-Rasoul M., Merritt R.E. (2022). National Trends and Outcomes of Segmentectomy in the Society of Thoracic Surgeons Database. Ann. Thorac. Surg..

[23] Yang M.Z., Tan Z.H., Li J.B., Xie C.L., Sun T.Y., Long H., Fu J.H., Zhang L.J., Lin P., Yang H.X. (2023). Comparison of Short-Term Outcomes Between Robot-Assisted and Video-Assisted Segmentectomy for Small Pulmonary Nodules: A Propensity Score-Matching Study. Ann. Surg. Oncol..

[24] Mao J., Tang Z., Mi Y., Xu H., Li K., Liang Y., Wang N., Wang L. (2021). Robotic and video-assisted lobectomy/segmentectomy for non-small cell lung cancer have similar perioperative outcomes: A systematic review and meta-analysis. Transl. Cancer Res..

[25] Echavarria M.F., Cheng A.M., Velez-Cubian F.O., Ng E.P., Moodie C.C., Garrett J.R., Fontaine J.P., Robinson L.A., Toloza E.M. (2016). Comparison of pulmonary function tests and perioperative outcomes after robotic-assisted pulmonary lobectomy vs segmentectomy. Am. J. Surg..

[26] Nguyen D., Gharagozloo F., Tempesta B., Meyer M., Gruessner A. (2019). Long-term results of robotic anatomical segmentectomy for early-stage non-small-cell lung cancer. Eur. J. Cardiothorac. Surg..

